# The prognosis of primary aldosteronism achieved incomplete surgical remission and changes in plasma aldosterone, plasma renin activity, and biochemical indicators

**DOI:** 10.5937/jomb0-54960

**Published:** 2025-09-05

**Authors:** Zihao Guo, Jing Wang, Xiang Ren, Xing Li, Yisheng Yin, Yiqun Tian, Zhenliang Qin, Kun Yuan, Xiaoyong Zeng

**Affiliations:** 1 Tongji Hospital, Tongji Medical College, Huazhong University of Science and Technology, Wuhan, China; 2 Institute of Organ Transplantation, Tongji Hospital, Tongji Medical College, Huazhong University of Science and Technology; Key Laboratory of Organ Transplantation, Ministry of Education; NHC Key Laboratory of Organ Transplantation; Key Laboratory of Organ Transplantation, Chinese Academy of Medical Sciences, Wuhan, China; 3 Department of Urology, Xijing Hospital, Air Force Medical University, Xi'an, China

**Keywords:** plasma aldosterone, plasma renin, nomogram, primary aldosteronism, surgical benefits, aldosteron u plazmi, renin u plazmi, nomo gram, primarni aldosteronizam, koristi od hirurškog zahvata

## Abstract

**Background:**

Some primary aldosteronism (PA) patients with spontaneous hypokalemia achieved incomplete remission after surgical treatment. In this study, we aimed to construct a nomogram to predict surgical benefits for primary aldosteronism (PA) patients with typical symptoms, incorporating changes in plasma aldosterone, plasma renin activity (PRA), and aldosterone/renin ratio (ARR) to help clinicians assess prognosis and develop optimised treatment plans.

**Methods:**

This retrospective cohort study enrolled 162 patients between January 2017 and January 2024. Baseline characteristics, clinical indicators, and biochemical results, including plasma aldosterone, PRA, ARR, and serum potassium, were compared among patients with different clinical and biochemical outcomes. A nomogram was developed and internally validated with risk factors selected from univariate and multivariate logistic regression analyses.

**Results:**

Complete clinical and biochemical success was achieved in 69 (42.6%) and 129 (79.6%). Five risk factors were used to develop a nomogram. The area under the receiver operating characteristic curve (AUC) was 0.856 (0.788-0.924) in the training dataset and 0.726 (0.580-0.872) in the validation dataset. The calibration curve showed good agreement, and the decision curve analysis demonstrated the clinical utility of this model.

**Conclusions:**

PA patients with older age, higher systolic blood pressure, lower plasma aldosterone, more than 5 years of hypertension, and an adrenal gland mass on the left side or both sides had more probability of achieving incomplete remission after the surgery.

## Abstract (sr)

**Uvod:**

Neki pacijenti sa primarnim aldosteronizmom (PA) i spontanom hipokalemijom postigli su nepotpunu remisiju nakon hirurškog lečenja. U ovoj studiji imali smo za cilj da konstruišemo nomogram za predviđanje koristi od hirurškog zahvata kod pacijenata sa primarnim aldosteronizmom (PA) i tipičnim simptomima, uzimajući u obzir promene u nivou aldosterona u plazmi, aktivnosti renina u plazmi (PRA) i odnos aldosteron/renin (ARR), kako bismo pomogli kliničarima u proceni prognoze i razvoju optimizovanih planova lečenja.

**Metode:**

Ova retrospektivna kohortna studija obuhvatila je 162 pacijenta između januara 2017. i januara 2024. godine. Osnovne karakteristike, klinički indikatori i biohemijski rezultati, uključujući nivo aldosterona u plazmi, PRA, ARR i serumskog kalijuma, upoređeni su među pacijentima sa različitim kliničkim i biohemijskim ishodima. Nomogram je razvijen i interno validiran pomoću faktora rizika odabranih na osnovu univarijantne i multivarijantne logističke regresione analize.

**Rezultati:**

Potpuni klinički i biohemijski uspeh postignut je kod 69 (42,6%) i 129 (79,6%) pacijenata. Za razvoj nomograma korišćeno je pet faktora rizika za nepotpunu remisiju. Površina ispod krive (AUC) bila je 0,856 (0,788-0,924) u trenažnom skupu podataka i 0,726 (0,580-0,872) u validacionom skupu podataka. Kriva kalibracije pokazala je dobro slaganje, a analiza odlučivanja potvrdila je kliničku upotrebljivost ovog modela.

**Zaključak:**

Pacijenti sa PA koji su stariji, imaju viši sistolni krvni pritisak, niži nivo aldosterona u plazmi, hipertenziju dužu od 5 godina i veću masu nadbubrežne žlezde sa leve strane ili obostrano, imaju veću verovatnoću da će postići nepotpunu remisiju nakon operacije.

## Introduction

Primary aldosteronism (PA), also known as Conn syndrome, is a pathological disease in which dysregulated and autonomous oversecretion of aldosterone leads to hypertension, hypokalemia, inhibition of plasma renin activity (PRA), and even cardiovascular and renal damage [1]. As a common cause of secondary hypertension, the prevalence of PA was positively correlated with hypertension grades [2]. Patients with PA had a higher cardiovascular risk compared with those with essential hypertension [3]. Therefore, to reduce the high risk that comes with PA, early diagnosis and suitable treatment are imperative.

Hypokalemia is another common symptom of PA, with a prevalence between 9 and 37% [4]. As the diagnostic capabilities of PA improved, many patients with PA had been detected before the onset of hypokalemia, making normokalemia and hypertensive PA the most prevalent type. However, studies have shown that hypokalemia increases the risk of cardiovascular events and target organ damage [5], which led to relatively poor prognoses for PA patients with spontaneous hypokalemia. As such, our study focused on PA patients with spontaneous hypokalemia and tried to find factors associated with poor prognoses.

The procedures of diagnosis, subtyping, and lateralisation of PA are complicated. After the aldosterone/renin ratio (ARR) screening test, at least one confirmatory test is required, of which the saline infusion test and the captopril challenge test are now commonly used in clinical practice. According to the Endocrine Society Clinical Practice Guideline, patients with spontaneous hypokalemia, PRA below the detectable threshold, and plasma aldosterone concentration (PAC) >20 ng/dL can be diagnosed with PA without confirmatory tests [6]. After determining the diagnoses, whether patients are treated by surgery or medication is decided by the subtype and laterality of PA based on adrenal computed tomography (CT) scans and adrenal vein sampling (AVS). Although AVS is the gold standard for determining tumour laterality, it’s limited to being performed in some large hospitals due to its invasive procedures, expensive costs, and high technical demands [7]. At the same time, thin-slice CT is widely adopted due to the development of its diagnostic performance. In a randomised controlled trial, Dekkers et al. [8] reported no significant differences in clinical benefits between treatments based on CT or AVS. The guideline also recommended that AVS may not be performed before unilateral adrenalectomy for patients under 35 years old with spontaneous hypokalemia, notably increased plasma aldosterone, and unilateral adrenal lesions compatible with cortical adenomas on adrenal CT characteristics [6].

Therefore, it is necessary to streamline diagnostic processes and explore optimised surgical indications. Through the observation of clinical practice, our team found that most patients with adrenal masses and typical PA symptoms, such as hypertension and hypokalemia, met the indications for surgery and had a good surgical prognosis, while some patients still suffered from partially relieved or persistent hypertension and/or hypokalemia. As such, we conducted this retrospective study to assess the prognosis of PA patients with adrenal masses and typical PA symptoms who underwent unilateral adrenal tumour resection. This study will help clinical doctors decide on treatment options and assess patients’ prognoses.

## Materials and methods

### Study design and patient selection

Aiming to develop and validate a nomogram to predict surgical benefits for PA patients, we collected Asian patient data between January 2017 and January 2024. The inclusion criteria were: 1) a history of hypertension and spontaneous hypokalemia; 2) imaging findings revealed features of aldo sterone producing adenoma (APA); 3) confirmed to be PA; 4) treated by unilateral laparoscopic adrenal tumour resection; 5) complete routine laboratory examinations; and 6) with follow-up for at least 6 months. The exclusion criteria were: 1) failed to undergo laparoscopic adrenal tumour resection or conducted several operations in parallel; 2) incomplete clinical record; 3) less than 6 months of followup; or 4) during pregnancy or lactation. Finally, we enrolled 162 patients in this retrospective study.

The Ethics Committee reviewed and approved the study protocol and abided by the principles of the Declaration of Helsinki. Informed consent was obtained before participants were enrolled.

### Data collection and definitions

Patient data was collected from the inpatient database. Clinical baseline characteristics included sex, age, family history of hypertension, history of diabetes, history of atrial fibrillation, preoperative blood pressure, known duration of hypertension, antihypertensive drug type and dosage, location and size of the tumour, and postoperative pathological findings. Biochemical test results included preoperative lowest serum potassium ion concentration, plasma aldosterone concentration (supine and erect position), and PRA (supine and erect position).

All patients underwent a follow-up for at least 6 months at the urology outpatient clinics or by telephone interviews collecting clinical and biochemical indications, including blood pressure, antihypertensive drugs, serum potassium ion concentration, plasma aldosterone concentration, and plasma renin activity.

According to the Primary Aldosteronism Surgical Outcome (PASO) criteria, patients’ clinical and biochemical outcomes were divided into complete, partial, and absent success, defined as remission, improvement, and persistence [9]. Hypertension and grading standards were based on the guidelines of the European Society of Hypertension [10]. Hypokalemia had the lowest serum potassium ion concentration, less than 3.5 mmol/L. The longest diameter describes tumour size.

### Statistical analysis

Since all quantitative variables were non-normally distributed and analysed by the Kolmogorov- Smirnov test, they were shown as medians with the first and third quartiles (Q1, Q3). Differences in non-normally distributed quantitative variables were compared with the Mann-Whitney U test for two independent groups, the Kruskal-Wallis H test for three or more independent groups, and the Wilcoxon matched-pairs signed rank test for self-controlled prepostoperative groups. Counts and percentages described categorical variables, and differences were compared using the Chi-square test or Fisher’s exact test.

Next, all patients were randomly divided into the training dataset (70%, n=113) and the internal validation dataset (30%, n=49). Variables were confirmed with no statistical difference between the training and validation datasets (Table 1). Then, we integrated patients who achieved complete clinical and biochemical success into one group, defined as complete remission, and the remaining patients into a separate group, defined as incomplete remission. Based on these two patient prognoses, univariate and multivariate logistic regression analyses were performed for factors with statistical significance or clinical meaning.

**Table 1:** Baseline characteristics. a. *p* values of less than 0.05 were considered significant.<br>b. Q1, the first Quartile; Q3, the third Quartile; ARR, aldosterone/renin ratio.

	Total (n=162)	Training set (n=113)	Validation set	*p*-value
Sex, male, n (%)	57 (35.2)	40 (35.4)	17 (34.7)	0.931
Age, median (Q1, Q3), years	52 (42, 59)	52 (41, 60)	51 (44, 59)	0.900
Age ≥60, n (%)	39 (24.1)	30 (26.6)	9 (18.4)	0.263
Preoperative BP, median (Q1, Q3), mmHg				
Systolic pressure	157 (145, 180)	155 (145, 180)	160 (145, 180)	0.774
Diastolic pressure	99 (87, 110)	97 (86, 110)	100 (90, 110)	0.285
Hypertension grade				0.882
grade 0	10 (6.2)	6 (5.3)	4 (8.2)	
grade 1	58 (35.8)	42 (37.2)	16 (32.7)	
grade 2	36 (22.2)	25 (22.1)	11 (22.5)	
grade 3	58 (35.8)	40 (35.4)	18 (36.7)	
Known duration of hypertension, median<br>(Q1, Q3), months	48 (12, 117)	36 (12, 120)	60 (12, 72)	0.885
Hypertension ≥5 years, n (%)	77 (47.5)	52 (46.0)	25 (51.0)	0.558
Numbers of antihypertensive drug types, n (%)				0.351
0	15 (9.3)	13 (11.5)	2 (4.1)	
1	61 (37.7)	40 (35.4)	21 (42.9)	
2	46 (28.4)	31 (27.4)	15 (30.6)	
3	35 (21.6)	24 (21.2)	11 (22.5)	
4	5 (3.1)	5 (4.4)	0 (0)	
Antihypertension drug types ＞2	40 (24.7)	29 (25.7)	11 (22.5)	0.663
Use of spironolactone, n (%)	18 (11.1)	10 (8.9)	8 (16.3)	0.164
Preoperative lowest serum potassium ion<br>concentration, median (Q1, Q3), mmol/L	3.21 (2.82, 3.39)	3.22 (2.80, 3.39)	3.20 (2.90, 3.33)	0.988
Plasma aldosterone (erect position),<br>median (Q1, Q3), pg/mL	159.5 (80.2, 368.8)	158.0 (93.2, 375.0)	160.0 (56.8, 331.0)	0.250
Plasma renin activity (erect position),<br>median (Q1, Q3), uIU/mL	2.6 (1.1, 6.2)	2.7 (1.1, 5.8)	2.4 (1.1, 7.3)	0.929
ARR (erect position), median (Q1, Q3), pg/uIU	58.6 (19.2, 186.1)	66.5 (19.3, 209.5)	49.6 (19.2, 158.8)	0.526
Plasma aldosterone (supine position),<br>median (Q1, Q3), pg/mL	170.5 (67.5, 379.3)	166.0 (76.6, 408.0)	184.6 (54.1, 365.0)	0.449
Plasma renin activity (supine position),<br>median (Q1, Q3), uIU/mL	1.6 (0.6, 3.9)	1.6 (0.6, 4.0)	1.2 (0.6, 3.8)	0.560
ARR (supine position), median<br>(Q1, Q3), pg/uIU	81.7 (28.1, 308.3)	83.1 (31.6, 292.7)	80.4 (22.4, 325.8)	0.662
Tumor size, median (Q1, Q3), mm	16 (13, 23)	17 (14, 24)	15 (12, 18)	0.013
Tumor laterality, n (%)				0.526
Left	81 (50.0)	58 (51.3)	23 (46.9)	
Right	53 (32.7)	34 (30.1)	19 (38.8)	
Bilateral	28 (17.3)	21 (18.6)	7 (14.3)	
Multiple tumor, n (%)	9 (5.6)	9 (8.0)	0 (0)	0.097
Diagnosis of diabetes, n (%)	16 (9.9)	13 (11.5)	3 (6.1)	0.443
History of atrial fibrillation, n (%)	3 (1.9)	3 (2.7)	0 (0)	0.554
Family history of hypertension, n (%)	65 (40.1)	50 (44.3)	15 (30.6)	0.104

Finally, a nomogram model was developed with potential factors to predict postoperative outcomes. The area under the receiver operating characteristic curve (AUC) was used to assess the model’s ability to discriminate between different patient outcomes. The calibration curve reflected the gap between the predicted and actual outcomes, and the Hosmer-Lemeshow test was also analysed to assess the goodness of fit. Based on the nomogram model-assisted decisions, the decision curve analysis evaluated the net benefits of surgical operations.

A two-sided P value<0.05 was considered to be statistically significant. Bonferroni’s correction adjusted the significance threshold for multiple hypotheses between three or more groups. All statistical analyses and data visualisation were conducted in SPSS Statistics (version 26.0) and GraphPad Prism (version 9.0). The nomogram model was constructed, and bootstrap resampling (1000 repetitions) was validated in R software (version 3.4.1) with the »rms and DecisionCurve« packages.

## Results

### Baseline characteristics and surgical benefits

A total of 162 Asian patients were included in this study, with their overall baseline characteristics presented in Table 1. This study included 57 males (35.2%) and 105 females (64.8%). The median age of patients was 52 (42, 59), with 39 patients (24.1%) ≥ 60 years old. Sixteen patients (9.9%) had a diagnosis of diabetes, nine (1.9%) had a history of atrial fibrillation, and sixty-five (40.1%) had a family history of hypertension. The median size of adrenal gland masses is 16 (13, 23) mm. As for tumour laterality, eighty-one patients (50.0%) were on the left side, fifty-three (32.7%) were on the right side, and the rest, twenty-eight (17.3%), were bilateral. Only 9 patients (5.6%) were found to have multiple tumours.

The median preoperative systolic blood pressure (SBP) was 157 (145, 180) mmHg, and diastolic blood pressure (DBP) was 99 (87, 110) mmHg, with 94 patients (58%) meeting hypertension grade 2 or 3. After a follow-up of 6~12 months (Table 2), the median of postoperative SBP and DBP was 135 (127, 143) mmHg and 77 (69, 85) mmHg, with only one patient (0.6%) meeting grade 2. There was a significant difference between pre-postoperative SBP and DBP, and the respective median of decreased blood pressure was 23 (11, 34) and 23 (13, 32) mmHg. Before the operation, 147 patients (90.7%) had taken antihypertensive drugs, whereas at the 6–12 months follow-up, 76 patients (46.9%) had no need to take antihypertensive drugs anymore, and 107 patients (66.6%) were taking fewer antihypertensive drugs.

**Table 2:** Pre-postoperative comparison of clinical and biochemical parameters. a. *p*-values of less than 0.05 were considered significant.<br>b. Q1, the first Quartile; Q3, the third Quartile; ARR, aldosterone/renin ratio.

	Preoperative	Postoperative	p-value
Blood pressure, median (Q1, Q3), mmHg			
Systolic pressure	157 (145, 180)	135 (127, 143)	**<0.001**
Diastolic pressure	99 (87, 110)	77 (69, 85)	**<0.001**
Hypertension grade, n (%)		<0.001	
Grade 0	10 (6.2)	107 (66.0)	
Grade 1	58 (35.8)	54 (33.3)	
Grade 2	36 (22.2)	1 (0.6)	
Grade 3	58 (35.8)	0 (0)	
Pre-post Delta systolic pressure, median (Q1, Q3), mmHg	23 (11, 34)		
Pre-post Delta diastolic pressure, median (Q1, Q3), mmHg	23 (13, 32)		
Numbers of antihypertensive drug types, n (%)		<0.001	
0	15 (9.3)	76 (46.9)	
1	61 (37.7)	60 (37.0)	
2	46 (28.4)	17 (10.5)	
3	35 (21.6)	7 (4.3)	
4	5 (3.1)	2 (1.2)	
Patients reduced antihypertension drug use, n (%)	107 (66.6)		
Lowest serum potassium ion concentration, median (Q1, Q3),	3.21 (2.82, 3.39)	3.98 (3.68, 4.29)	**<0.001**
Plasma aldosterone (erect position), median (Q1, Q3), pg/mL	159.5 (79.5, 370.0)	74.4 (56.5, 90.8)	**<0.001**
Plasma renin activity (erect position), median (Q1, Q3), uIU/mL	2.6 (1.1, 6.2)	7.7 (5.1, 14.7)	**<0.001**
ARR (erect position), median (Q1, Q3), pg/uIU	58.6 (19.2, 188.8)	8.8 (5.6, 13.9)	**<0.001**

As a condition of enrollment, hypokalemia was significantly improved. The median of the lowest serum potassium ion concentration before and after the operation was 3.21 (2.28, 3.39) and 3.98 (3.68, 4.29) mmol/L. However, there were still 16 patients (9.9%) who showed hypokalemia after surgery. Significant differences were observed in plasma aldosterone, PRA, ARR, and serum potassium before and after the operation. Plasma aldosterone levels decreased from 159.5 (79.5, 370.0) to 74.4 (56.5, 90.8) pg/mL (p<0.001), PRA increased from 2.6 (1.1, 6.2) to 7.7 (5.1, 14.7) uIU/mL (p<0.001), and ARR decreased from 58.6 (19.2, 188.8) to 8.8 (5.6, 13.9) pg/uIU (p<0.001). Similarly, serum potassium levels improved significantly, rising from 3.21 (2.82, 3.39) to 3.98 (3.68, 4.29) mmol/L (p<0.001). These improvements were consistent across all patient subgroups, further validating the clinical utility of these biochemical markers.

**Figure 1:**
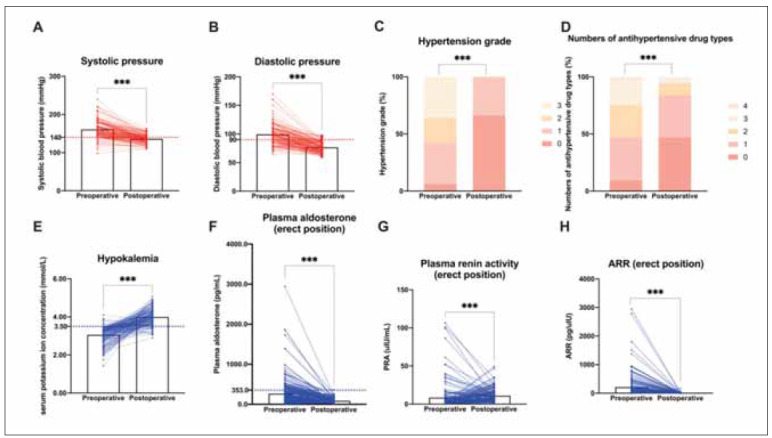
Pre-postoperative comparison of clinical and biochemical parameters, including (A- H): systolic blood pressure, diastolic blood pressure, hypertension grade, numbers of antihypertensive drug types, serum potassium ion concentration, plasma aldosterone(erect position), PRA (erect position), and ARR (erect position).<br>Note: ***: p<0.001; PRA: plasma renin activity; ARR: aldosterone/renin ratio.

The comparison of clinical and biochemical indicators was self-controlled, visualised in Figure 1. It was evident that most of the PA patients with typical features could benefit from unilateral laparoscopic adrenal tumour resection.

### Factors associated with clinical and biochemical outcomes

After 6–12 months of follow-up, complete clinical success was achieved in 69 patients (42.6%), improvement in blood pressure management (partial clinical success) was seen in 81 patients (50.0%), and absent clinical success was found in 12 patients (7.4%). The proportion of patients attaining complete biochemical success was much higher than that of patients achieving complete clinical success. A hundred and twenty-nine patients (79.6%) achieved complete biochemical success, only nine (5.6%) showed partial biochemical success, and twenty-four (14.8%) got absent biochemical success. By comparing clinical indicators and biochemical test results, some potential factors significantly differed between patients with different outcomes. For patients with different clinical outcomes, we identified 15 out of 24 variables statistically different, including age, age ≥60, SBP, hypertension grade, known duration of hypertension, numbers of antihypertensive drug types, plasma aldosterone (supine and erect position), plasma renin activity (supine and erect position), ARR (supine and erect position), tumour size, and laterality (Table 3). As for patients with different biochemical outcomes, fewer variables were statistically different, including the lowest serum potassium ion concentration, plasma aldosterone (supine and erect position), and tumour laterality (Table 4).

**Table 3:** Characteristics of patients, stratified by clinical outcome. a. *p* values of less than 0.05 were considered significant.<br>b. Q1, the first Quartile; Q3, the third Quartile; NA, not applicable; BP, blood pressure; ARR, aldosterone/renin ratio.

	Clinical outcome			Overall<br>*p*-value	Pairwise comparison (p values)		
	Complete<br>success	Partial<br>success	Absent<br>success		Complete<br>vs. partial	Partial<br>vs. absent	Complete<br>vs. absent
Patients number, n (%)	69 (42.6)	81 (50.0)	12 (7.4)	NA	NA	NA	NA
Sex, male, n (%)	23 (33.3)	27 (33.33)	7 (58.33)	0.218	NA	NA	NA
Age, median<br>(Q1, Q3), years	45 (38, 54)	54 (46, 60)	62 (56, 65)	**<0.001**	**<0.001**	0.105	**0.001**
Age ≥ 60, n (%)	6 (8.7)	25 (30.9)	8 (66.7)	**<0.001**	**<0.001**	0.036	**<0.001**
Preoperative BP, median<br>(Q1, Q3), mmHg							
Systolic pressure	153 (140, 170)	160 (147, 180)	163 (154, 180)	0.041	0.039	0.047	0.358
Diastolic pressure	100 (87, 110)	99 (89, 110)	93 (84, 105)	0.745	NA	NA	NA
Hypertension grade				**0.014**	**0.001**	0.769	0.249
grade 0	10 (14.5)	0 (0)	0 (0)				
grade 1	22 (31.9)	32 (39.5)	4 (33.3)				
grade 2	13 (18.8)	19 (23.5)	4 (33.3)				
grade 3	24 (34.8)	30 (37.0)	4 (33.3)				
Known duration of<br>hypertension, median<br>(Q1, Q3), months	24 (10, 48)	72 (24, 120)	75 (9, 144)	**<0.001**	**<0.001**	0.647	0.107
Hypertension ≥5 years, n (%)	17 (24.6)	53 (65.4)	7 (58.3)	**<0.001**	**<0.001**	0.876	0.044
Numbers of antihypertensive<br>drug types, n (%)				**0.001**	**0.004**	**0.005**	0.033
0	9 (13.0)	3 (3.7)	3 (25.0)				
1	32 (46.4)	25 (30.9)	4 (33.3)				
2	16 (23.2)	30 (37.0)	0 (0)				
3	12 (17.4)	18 (22.2)	5 (41.7)				
4	0 (0)	5 (6.2)	0 (0)				
Antihypertension drug types<br>＞2	12 (17.4)	23 (28.4)	5 (41.7)	0.109	NA	NA	NA
Use of spironolactone, n (%)	8 (11.6)	9 (11.1)	1 (8.3)	0.946	NA	NA	NA
Preoperative lowest serum<br>potassium ion<br>concentration, median<br>(Q1, Q3), mmol/L	3.10<br>(2.77, 3.38)	3.25<br>(2.89, 3.40)	2.97<br>(2.69, 3.35)	0.278	NA	NA	NA
Plasma aldosterone<br>(erect position), median<br>(Q1, Q3), pg/mL	276.0<br>(123.0, 430.0)	121.0<br>(63.7, 258.0)	127.0<br>(65.0, 154.8)	**<0.001**	**<0.001**	0.399	**0.007**
Plasma renin activity<br>(erect position), median<br>(Q1, Q3), uIU/mL	2.1<br>(0.9, 4.2)	2.9<br>(1.1, 8.9)	4.7<br>(2.5, 7.6)	**0.038**	0.030	0.434	0.056
ARR (erect position), median<br>(Q1, Q3), pg/uIU	150.0<br>(35.7, 253.3)	33.5<br>(9.2, 128.8)	31.1<br>(10.0, 48.8)	**<0.001**	**<0.001**	0.442	**0.005**
Plasma aldosterone<br>(supine position), median<br>(Q1, Q3), pg/mL	299.0<br>(112.0, 463.0)	111.0<br>(52.2, 271.0)	98.7<br>(59.0, 168.8)	**<0.001**	**<0.001**	0.369	**0.004**
Plasma renin activity<br>(supine position), median<br>(Q1, Q3), uIU/mL	1.1<br>(0.5, 1.9)	2.3<br>(0.7, 5.1)	3.7<br>(1.2, 6.5)	**0.005**	**0.003**	0.482	0.026
ARR (supine position),median<br>(Q1, Q3), pg/uIU	203.8<br>(67.0, 563.8)	56.0<br>(14.0, 105.8)	42.8 (7.6, 86.7)	<0.001	<0.001	0.411	0.001
Tumor size, median<br>(Q1, Q3), mm	15 (12, 20)	18 (14, 28)	16 (14, 19)	0.027	0.009	0.246	0.818
Tumor laterality, n (%)				**<0.001**	**<0.001**	**0.010**	**<s0.001**
Left	33 (47.8)	45 (55.6)	3 (25.0)				
Right	34 (49.3)	18 (22.2)	1 (8.3)				
Bilateral	2 (2.9)	18 (22.2)	8 (66.7)				
Multiple tumor, n (%)	3 (4.4)	4 (4.9)	2 (16.7)	0.229			
Diagnosis of diabetes, n (%)	5 (7.3)	10 (12.4)	1 (8.3)	0.570	NA	NA	NA
History of atrial fibrillation,<br>n (%)	0 (0)	3 (3.7)	0 (0)	0.405	NA	NA	NA
Family history of hypertension,<br>n (%)	24 (34.8)	35 (43.2)	6 (50.0)	0.443	NA	NA	NA

**Table 4:** Characteristics of patients, stratified by biochemical outcome. a.* p* values of less than 0.05 were considered significant.<br>b. Q1, the first Quartile; Q3, the third Quartile; NA, not applicable; BP, blood pressure; ARR, aldosterone/renin ratio.

	Biochemical outcome			Overall<br>*p*-value	Pairwise comparison (p values)		
	Complete<br>success	Partial<br>success	Absent<br>success		Complete<br>vs. partial	Partial vs.<br>absent	Complete<br>vs. absent
Patients number, n (%)	129 (79.6)	9 (5.6)	24 (14.8)	NA	NA	NA	NA
Sex, male, n (%)	47 (36.4)	2 (22.2)	8 (33.3)	0.675	NA	NA	NA
Age, median (Q1, Q3), years	51 (41, 59)	44 (41, 50)	55 (50, 63)	0.068	NA	NA	NA
Age ≥60, n (%)	29 (22.5)	2 (22.2)	8 (33.3)	0.516	NA	NA	NA
Preoperative BP, median<br>(Q1, Q3), mmHg							
Systolic pressure	155 (144, 180)	163 (160, 180)	160 (148, 178)	0.597	NA	NA	NA
Diastolic pressure	99 (88, 110)	101 (100, 110)	91 (86, 112)	0.649	NA	NA	NA
Hypertension grade				0.353	NA	NA	NA
grade 0	9 (7.0)	1 (11.1)	0 (0)				
grade 1	47 (36.4)	1 (11.1)	10 (41.7)				
grade 2	26 (20.2)	4 (44.4)	6 (25.0)				
grade 3	47 (36.4)	3 (33.3)	8 (33.3)				
Known duration of hypertension,<br>median (Q1, Q3), months	36 (12, 84)	120 (60, 120)	66 (12, 120)	0.132	NA	NA	NA
Hypertension ≥5 years, n (%)	57 (44.19)	7 (77.78)	13 (54.17)	0.119	NA	NA	NA
Numbers of antihypertensive<br>drug types, n (%)				0.991	NA	NA	NA
0	13 (10.1)	0 (0)	2 (8.3)				
1	49 (38)	3 (33.3)	9 (37.5)				
2	36 (27.9)	3 (33.3)	7 (29.2)				
3	27 (20.9)	3 (33.3)	5 (20.8)				
4	4 (3.1)	0 (0)	1 (4.2)				
Antihypertension drug types ＞2	31 (24.0)	3 (33.3)	6 (25.0)	0.822	NA	NA	NA
Use of spironolactone, n (%)	15 (11.6)	1 (11.1)	2 (8.3)	1.000	NA	NA	NA
Preoperative lowest serum<br>potassium ion concentration,<br>median (Q1, Q3), mmol/L	3.22<br>(2.86, 3.39)	2.39<br>(2.30, 2.87)	3.25<br>(3.00, 3.41)	**0.014**	0.629	0.017	**<0.001**
Plasma aldosterone<br>(erect position), median<br>(Q1, Q3), pg/mL	160.0<br>(80.2, 373.0)	418.0<br>(186.0, 517.0)	133.0<br>(66.9, 214.3)	**0.015**	**0.014**	**0.004**	0.199
Plasma renin activity<br>(erect position), median<br>(Q1, Q3), uIU/mL	2.6<br>(1.1, 6.2)	2.3<br>(1.0, 3.8)	2.8<br>(1.0, 10.8)	0.869	NA	NA	NA
ARR (erect position), median<br>(Q1, Q3), pg/uIU	60.8<br>(19.1, 194.4)	93.5<br>(66.5, 464.4)	47.7<br>(13.6, 96.5)	0.119	NA	NA	NA
Plasma aldosterone<br>(supine position), median<br>(Q1, Q3), pg/mL	168.0<br>(68.4, 408.0)	365.0<br>(227.0, 559.0)	129.5<br>(48.9, 247.4)	**0.022**	0.034	**0.006**	0.120
Plasma renin activity<br>(supine position), median<br>(Q1, Q3), uIU/mL	1.4<br>(0.6, 3.8)	1.1<br>(0.5, 4.0)	2.3<br>(1.2, 4.7)	0.639	NA	NA	NA
ARR (supine position),<br>median (Q1, Q3), pg/uIU	83.2<br>(30.3, 308.4)	349.4<br>(55.2, 897.3)	69.8<br>(21.1, 90.1)	0.077	NA	NA	NA
Tumor size, median<br>(Q1, Q3), mm	16 (12, 22)	18 (13, 21)	19 (14, 27)	0.635	NA	NA	NA
Tumor laterality, n (%)				**0.003**	0.032	0.894	**0.006**
Left	66 (51.2)	4 (44.4)	11 (45.8)				
Right	48 (37.2)	1 (11.1)	4 (16.7)				
Bilateral	15 (11.6)	4 (44.4)	9 (37.5)				
Multiple tumor, n (%)	8 (6.2)	0 (0)	1 (4.2)	1.000	NA	NA	NA
Diagnosis of diabetes, n (%)	12 (9.3)	1 (11.1)	3 (12.5)	0.775	NA	NA	NA
History of atrial fibrillation, n (%)	1 (0.8)	1 (11.1)	1 (4.2)	0.055	NA	NA	NA
Family history of hypertension, n (%)	48 (37.2)	5 (55.6)	12 (50.0)	0.313	NA	NA	NA

Univariate and multivariate logistic regression analyses were used to identify independent predictive factors associated with patient outcomes (Table 5). Univariate analysis suggested age (OR:1.07, 95%CI:1.03–1.11, P<0.001), SBP (OR:1.02, 95% CI:1.01–1.04, P=0.047), duration of hypertension <5 years (OR:0.12, 95% CI:0.05–0.29, P<0.001), plasma aldosterone at erect position (OR:0.99, 95%CI: 0.99–0.99, P=0.006), the tumour on the right side (OR:0.31, 95%CI: 0.13–0.75, P=0.009), and bilateral tumours (OR:5.39, 95%CI: 1.14–25.46, P=0.033) were potential predictors of patient outcomes. Factors with significant differences in the univariate logistic regression analysis were included in the multivariate regression analysis, the latter of which found out that patients with lower SBP (OR:1.03, 95%CI:1.01–1.05, P=0.036), duration of hypertension<5 years (OR:0.15, 95%CI: 0.05–0.49, P=0.002), higher plasma aldosterone at erect position (OR:0.99, 95%CI: 0.99–0.99, P=0.038), and the tumour on the right side were more likely to achieve complete remission. The low predictive efficacy of plasma aldosterone at the erect position, though a statistically significant predictor, might be related to its units, for each unit increase in plasma aldosterone having a relatively small effect on patient outcomes.

**Table 5:** Logistic regression analysis of factors associated with clinical and biochemical outcomes. a. *p* values of less than 0.05 were considered significant.<br>b. OR: odds ratio; CI, Confidence Interval; SBP, systolic blood pressure; ARR, aldosterone/renin ratio.

	Univariate analysis		Multivariate analysis	
	OR (95%CI)	*p*-value	OR (95%CI)	*p*-value
Sex (Female)	0.73 (0.33–1.62)	0.439		
Age (per 1 year old)	1.07 (1.03–1.11)	**<0.001**	1.03 (0.98–1.09)	0.208
SBP (per 1mmHg)	1.02 (1.01–1.04)	**0.047**	1.03 (1.01–1.05)	**0.036**
Hypertension <5 years	0.12 (0.05–0.29)	**<0.001**	0.15 (0.05–0.49)	**0.002**
Antihypertensive drug types 2	0.39 (0.15–0.99)	0.050		
Serum potassium ion concentration (per 1mmol/L)	1.32 (0.58–3.04)	0.508		
Plasma aldosterone (erect position, per 1pg/mL)	0.99 (0.99–0.99)	**0.006**	0.99 (0.99–0.99)	**0.038**
ARR (erect position, per 1pg/uIU)	1.00 (1.00–1.00)	0.063		
Tumor size (per 1mm)	1.00 (0.97–1.03)	0.972		
Tumor laterality				
Left	1.00 (Reference)		1.00 (Reference)	
Right	0.31 (0.13–0.75)	**0.009**	0.33 (0.11–0.99)	**0.048**
Bilateral	5.39 (1.14–25.46)	**0.033**	2.92 (0.51–16.57)	0.227

### Development and validation of the nomogram

**Figure 2:**
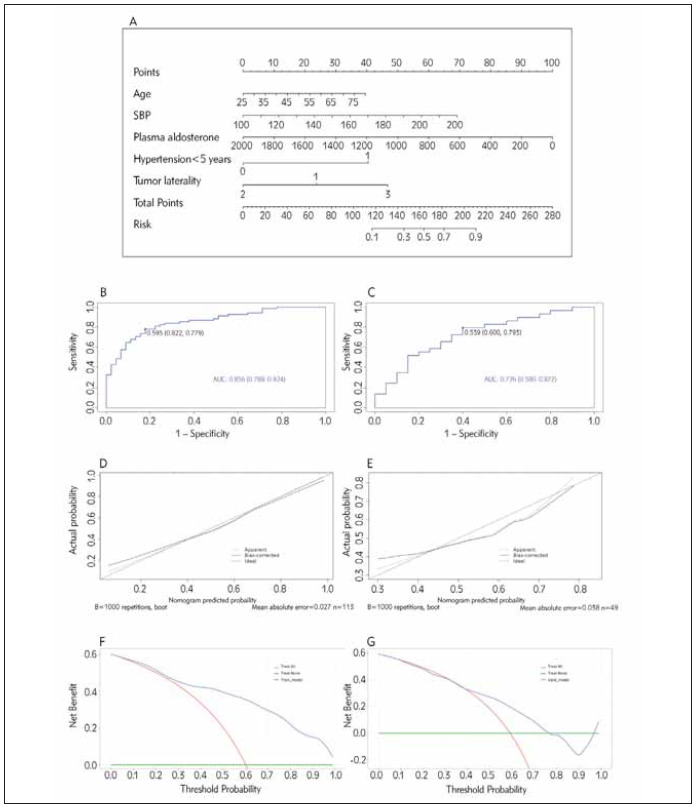
(A) A nomogram to predict surgical benefits for typical PA patients. The sum of points gained from each factor is projected vertically to the risk axis, and the value on the risk axis is the probability of achieving incomplete remission after surgery.<br>(Note: Age: years old; SBP: mmHg; Plasma aldosterone: erect position, pg/mL; Hypertension <5 years: 0 for true, 1 for false; Tumor laterality: 1 for the left side of the adrenal gland, 2 for the right side, 3 for two sides.) (B–C) The ROC curve with an AUC of 0.856 (0.788–0.924) of the training dataset (B) and with an AUC of 0.726 (0.580–0.872) of the validation dataset (C) shows good predictive accuracy and discrimination ability. (D–E) The training dataset (D) and the validation dataset (E) calibration curve. The X-axis shows the nomogrampredicted probability, and the Y-axis shows the actual probability of incomplete remission. (F–G) The decision curve analysis indicates that patients can benefit from using the nomogram prediction model in the training dataset (F) and the validation dataset (G).<br>Note: PA: primary aldosteronism; SBP: systolic blood pressure; ROC: the receiver operating characteristic curve; AUC: The area under ROC; B, boot: bootstrap.

A nomogram was constructed with the training dataset and evaluated with the internal validation dataset (Figure 2A). The sum of points gained from each factor was projected vertically onto the risk axis as the probability of achieving a poor prognosis (incomplete remission).

The ROC curve demonstrated the changing relationship between sensitivity and specificity with an AUC of 0.856 (0.788–0.924) in the training dataset (Figure 2B) and an AUC of 0.726 (0.580–0.872) in the validation dataset (Figure 2C), indicating good accuracy in predicting and discriminating patients with different outcomes. The calibration curve showed good consistency between predicted and actual probabilities in the training dataset (Figure 2D) and moderate calibration in the validation dataset (Figure 2E). In addition, the P values of the Hosmer-Lemeshow test were 0.961 and 0.391 in the training and validation datasets, which also suggested good agreement with the nomogram model. The decision curve analysis indicated that patients going through operations based on the training model prediction led to a higher net benefit than those treated with all or none, except for a small range of low threshold probability (Figure 2F). Patients treated based on the validation model gained a higher net benefit when the threshold probability ranged from about 0.40 to 0.75 (Figure 2G).

## Discussion

In this study, PA patients with hypertension and spontaneous hypokalemia were enrolled to assess surgical benefits, identify factors related to prognosis, and construct a nomogram model to predict surgical outcomes. After the unilateral laparoscopic adrenal tumour resection treatment, 92.6% of patients achieved remission or improvement in hypertension, and 85.2% had complete or partial success in biochemical outcomes, suggesting that most of these patients benefited from surgery. Moreover, we still need to screen and predict each patient’s prognosis in clinical practice. Variables that significantly differed among patients with different outcomes showed potential for prediction, five of which were identified as independent factors and were incorporated into the nomogram model. All predictive factors were easily obtainable and non-invasive, involving clinical characteristics, biochemical results, and radiological features, showing that our model assessed patients in multiple dimensions.

Recent studies have further elucidated factors influencing surgical outcomes in primary aldosteronism (PA) patients. A comprehensive international consensus analysed remission rates post-adrenalectomy, identifying variables such as age, duration of hypertension, and preoperative systolic blood pressure as significant predictors of clinical outcomes. These findings align with our study, reinforcing the importance of these factors in prognostic assessments. Additionally, the development of nomograms in other medical fields has demonstrated their utility in predicting patient outcomes. For instance, a nomogram that predicted survival in hepatocellular carcinoma patients undergoing transarterial chemoembolisation showed higher predictive accuracy than traditional prognostic models. This underscores the potential of nomogram models, like ours, to enhance individualised patient care by integrating multiple prognostic factors.

Furthermore, the role of hypokalemia in PA prognosis has been highlighted in recent literature. Studies have shown that spontaneous hypokalemia is often associated with more severe disease phenotypes and may predict poorer surgical outcomes. This emphasises the need for careful preoperative assessment of serum potassium levels, as incorporated in our nomogram model.

These studies corroborate our findings and support the clinical application of our nomogram model in predicting surgical outcomes for PA patients. By considering a combination of easily obtainable clinical and biochemical parameters, our model offers a practical tool for personalised treatment planning, ultimately aiming to improve patient outcomes.

In previous studies, multiple factors were included in nomogram models in different combinations. Unlike most models focused on distinguishing APA and idiopathic hyperaldosteronism, while the presence of microadenoma or nonfunctioning adenoma interfered with the accuracy of model predictions, our model focused on predicting a patient’s surgical prognosis, which directly helps clinical doctors decide treatment plans and focus on high-risk patients.

Hypertension, the most common symptom of PA, showed major predictive value, as two associated indicators were incorporated in the nomogram model. Our model demonstrated that incomplete remission was more likely to happen in patients with older age, higher SBP, and a longer duration of hypertension. Patients with those factors also had a higher risk of primary hypertension, which made PA with primary hypertension the most common reason for patients having absent clinical success [11]. According to PASO, age, sex, and number of antihypertensive medications were factors related to complete clinical success [9]. However, in our study, hypertensive drugs over two types were not an independent factor of incomplete remission. Moreover, sex didn’t show statistical differences among groups of different outcomes, along with a family history of hypertension, a history of atrial fibrillation, and a history of spironolactone use. The reason for this result might be that the sample size of our study was not adequate.

The onset of spontaneous hypokalemia was often associated with a poor prognosis [5]. As such, our study set this condition as one of the inclusion criteria. Then, we found out that the preoperative lowest serum potassium ion concentrations were statistically different in patients with different biochemical outcomes, while which were not independent risk factors for incomplete remission.

James et al. [12] found that excess aldosterone and hypokalemia were associated with insulin resistance, which led to metabolic syndromes like diabetes. But, our study showed no statistical difference in diabetes among groups of different outcomes.

Lateralisation of PA was important for the treatment strategy. A study showed that based on CT and AVS, APA and nonfunctioning tumours were more likely to develop on the left adrenal gland [13]. In our study, the right side was a protective factor for prognosis compared with tumours on the left side, while bilateral masses were a risk factor for incomplete remission. For patients with bilateral masses on adrenal glands, we only performed unilateral resection for those with a notably larger mass on one of the sides. As such, this result might be due to the misdiagnosis of CT-based lateralisation. Also, we measured the size of masses based on CT scans, which showed statistical significance among different clinical outcomes but were not a risk factor for incomplete remission according to the logistic regression analysis.

Unlike ARR, which showed good PA screening performance, plasma aldosterone was separately included in our nomogram model as a predictor, which also achieved the highest scores. Notably, the axis of plasma aldosterone is in descending order, indicating that the higher plasma aldosterone may get more excellent surgical benefits. Long-lasting excessive aldosterone in plasma can cause inflammatory, oxidative, and fibrotic effects in the heart, kidney, and blood vessels, which also lead to cardiovascular disease and kidney dysfunction like heart failure, arterial fibrillation, and chronic kidney disease [14]. Early diagnosis and surgical treatment will ameliorate excessive plasma aldosterone and improve patients’ prognoses.

There were several limitations in this study. First, our data was collected from a single centre and validated with an internal dataset, which lacks external validation from other clinical centres. Second, the sample size of our study was insufficient and, therefore, needs to be expanded to obtain more reliable conclusions. Third, retrospective studies inevitably had inherent biases, such as information bias. Thus, a multicenter prospective study with a larger sample size should be performed. At last, we only enrolled patients who had received surgery, and the treatment plans were based on CT scans without AVS, which might cause selection bias [15].

## Conclusion

In this study, we developed a practical nomogram to predict surgical outcomes for primary aldosteronism (PA) patients with hypertension and spontaneous hypokalemia. The nomogram incorporates key clinical and biochemical predictors, including plasma aldosterone, plasma renin activity, aldosterone/renin ratio (ARR), and tumour laterality, offering a multidimensional approach to assess surgical prognosis. The findings underscore the utility of this tool in aiding clinicians in identifying high-risk patients likely to achieve incomplete remission and making informed surgical decisions. By integrating easily accessible and non-invasive parameters, the nomogram facilitatespersonalised treatment planning, improving patient outcomes and resource allocation in clinical settings. Future studies should focus on external validation in larger, multicenter cohorts to enhance its generalizability and impact.

## Dodatak

### List of abbreviations

PA, primary aldosteronism;<br>PRA, plasma renin activity;<br>ARR, aldosterone/renin ratio;<br>PAC, plasma aldosterone concentration;<br>CT, computed tomography;<br>AVS, adrenal vein sampling;<br>PASO, primary aldosteronism surgical outcome;<br>Q1, Q3, the first and third quartiles;<br>ROC, the receiver operating characteristic curve;<br>AUC, the area under receiver operating characteristic curve;<br>SBP, systolic blood pressure;<br>DBP, diastolic blood pressure;<br>APA, aldosterone producing adenoma;<br>OR, odds ratio;<br>CI, confidence interval.

### Acknowledgements

We thank all participants and the Department of Medical Records of Tongji Hospital for providing the original data.

### Funding

This study was supported by the National Natural Science Foundation of China (grant number NSFC #82070715).

### Author contribution

Research conception and design: ZH Guo, J Wang and XY Zeng. Data acquisition: ZH Guo, ZL Qin, K Yuan and YQ Tian. Data analysis and interpretation: ZH Guo, YS Yin and X Ren. Drafting of the manuscript: ZH Guo and X Li. Critical revision of the manuscript: XY Zeng.

### Ethics statements

The present study protocol was reviewed and approved by the Ethics Committee of Huazhong University of Science and Technology (approval number: TJ-IRB20231277) and abided by the principles of the Declaration of Helsinki. Informed consent was obtained before participants were enrolled.

### Conflict of interest statement

All the authors declare that they have no conflict of interest in this work.
